# A case of vasculitis with digital necrosis secondary to cryoglobulinemia

**DOI:** 10.1016/j.jdcr.2025.11.012

**Published:** 2025-11-19

**Authors:** Pratiksha Patra, Kalie Nuss, Laurie A. Temiz, Neel K. Shah, Ann Lin

**Affiliations:** a Department of Internal Medicine, University of South Florida, Tampa, Florida; b University of South Florida Morsani College of Medicine, Tampa, Florida; c Department of Dermatology & Cutaneous Surgery, University of South Florida, Tampa, Florida

**Keywords:** cryoglobulinemia, digital necrosis, vasculitis

## Introduction

Digital necrosis is suggestive of a wide variety of etiologies, including vascular, inflammatory, malignant, neurotropic, infectious, and iatrogenic causes. One less common cause of digital necrosis, cryoglobulinemia (CG), refers to the presence of circulating cryoglobulin proteins that either deposit as immune complexes in small-to-medium-sized blood vessels (types II and III) or cause vascular occlusion through hyperviscosity (type I). Although CG is typically managed by hematologists and oncologists, its integumentary manifestations are relevant to dermatologists as well. The case presented here serves to describe 1 instance where CG presented primarily as digital necrosis, thereby raising awareness to the disease as a causative agent of dermatologic complaints and alerting dermatologists to its implications within their field.

## Case presentation

**Fig 1:**
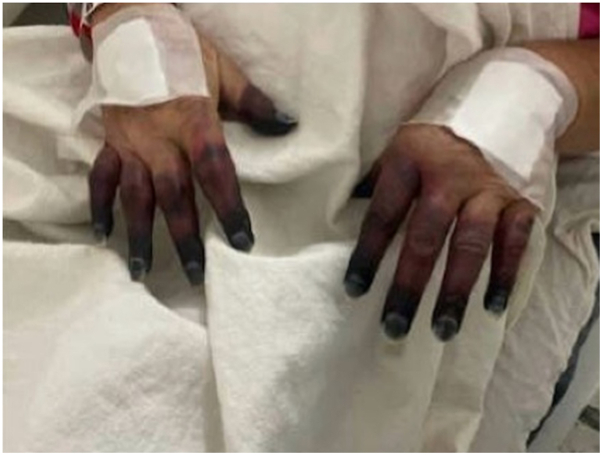
Bilateral distal digital necrosis of the hands progressing proximally.

**Fig 2:**
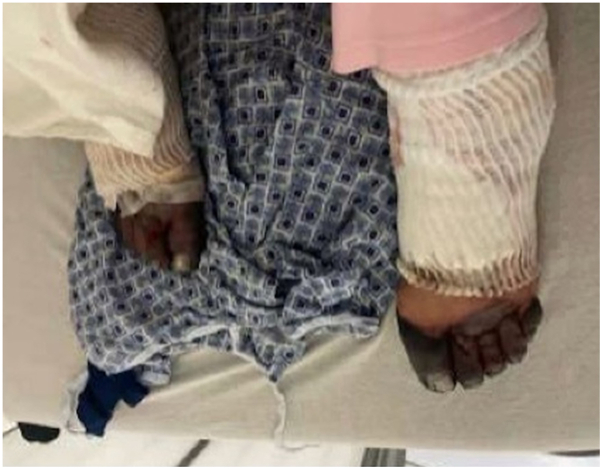
Bilateral digital necrosis of the feet.

A 75-year-old female presented to the emergency department with a progressive purpuric rash on her bilateral legs and necrosis of her bilateral fingers and toes. She first noticed small blisters on her toes 1 month prior, which she believed were caused by ant bites. Her primary care physician prescribed her a 10-day course of Bactrim to empirically treat for infection; however, the blisters persisted and spread proximally. Eventually, her fingers and toes became discolored and sclerotic. Four weeks later, she presented to an off-site hospital, where she was given several antibiotic therapies including ceftriaxone, vancomycin, cefalexin, and doxycycline. Rheumatology and hematology were consulted, and a skin biopsy was performed. The patient was subsequently transferred to this hospital’s emergency department, where she was administered vancomycin and cefepime and admitted for worsening necrosis of her fingers and toes (Figs 1 and 2). Her workup included bilateral Doppler ultrasound, ankle-brachial indices, and x-rays of the bilateral hands and feet, which revealed only minor arterial calcification and a displaced left calcaneal fracture. Laboratories were significant for positive hepatitis C autoreactivity, antinuclear antibody, and Sjögren's syndrome antibody SS-A, elevated erythrocyte sedimentation rate, gamma globulin gap (gamma gap), immunoglobulin G (IgG), white blood cell count, aspartate aminotransferase, and alanine aminotransferase, and low complement levels of C3 and C4. Given the pattern of these laboratory abnormalities and blood cultures returning negative with no other signs of systemic infection, concern for an autoimmune etiology arose. During her stay at the hospital, laboratory results from the off-site hospital revealed positive cryoglobulins and IgG lambda monoclonal paraprotein, and skin biopsy demonstrated focal ischemic necrosis with leukocytoclastic vasculitis and small vessel thrombosis, consistent with CG. As a result, high-dose methylprednisolone was initiated. Unfortunately, during her inpatient stay, she had a diverticular rupture, which led to sepsis secondary to Enterococcus. She expired 8 days after starting treatment.

## Discussion

Digital necrosis is an uncommon presentation with a broad differential. Vascular causes include arterial insufficiency, thromboembolism, and thromboangiitis obliterans, among others. Autoimmune conditions such as systemic lupus erythematosus and Sjögren’s syndrome may also cause digital necrosis.^1^ Other etiologies include infectious, hematologic, and traumatic origins. Several sources even recognize it as a rare manifestation of carpal tunnel syndrome, multiple myeloma, and COVID-19.^2^, ^3^, ^4^ More generally, digital necrosis in females is suggestive of connective tissue pathology, whereas in males, it is suggestive of arteriopathy.^4^ Digital necrosis is a conspicuous manifestation of disease with debilitating consequences, which makes rapid diagnosis and treatment imperative.

CG involves the presence of abnormal proteins (cryoglobulins) in the blood that precipitate at cold temperatures, leading to vasculitis and small vessel occlusion. It is often associated with autoimmune diseases and chronic infections, like hepatitis C virus. CG typically presents with a classic triad of purpura, arthralgia, and weakness.^5^^,^^6^ There are 3 types of CG, including type 1 (T1), which is monoclonal and associated with lymphoproliferative disorders, and mixed CG (MC), which includes both monoclonal (type 2) and polyclonal (type 3) forms, the former of which has a higher association with hepatitis C virus^5^ (Table I). CG is often diagnosed based on characteristic clinical features and the detection of cryoglobulins in serum. Histopathologic findings vary by subtype: type I typically demonstrates an occlusive vasculopathy, whereas types II and III more often show features of leukocytoclastic vasculitis, including fibrinoid necrosis of vessel walls, neutrophilic infiltration with leukocytoclastic debris, and perivascular inflammation.^7^ Skin manifestations may present in cases of CG, and they may differ with CG type. The dermatologic symptoms of T1 tend to relate to hyperviscosity, with some of the most common manifestations including Raynaud’s phenomenon, distal gangrene, livedo reticularis, acrocyanosis, and purpura.^5^ Both T1 and MC may present with digital necrosis, and the most common symptom, palpable purpura, may be accompanied by ulcers, which can lead to necrosis and sepsis.^5^

**Table I:** Comparison of T1 and MC cryoglobulinemia

	T1	MC
Type of cryoglobulin	Monoclonal IgG or IgM^5^	Polyclonal IgG with monoclonal IgM (type 1) or with polyclonal IgM (type 2)^8^
Classic symptoms	Triad of mucosal bleeding, visual and neurologic changes, and acral necrosis related to hyperviscosity^5^^,^^8^	Triad of purpura, arthralgia, and myalgia^5^^,^^6^
Cause of skin manifestations	Vascular obstruction^5^	Immune complex-mediated vasculitis^5^
Histopathology	Occlusive vasculopathy, typically without leukocytoclastic vasculitis^7^	Leukocytoclastic vasculitis with fibrinoid necrosis, neutrophils, and leukocytoclastic debris^7^
Associated disorders	Underlying lymphoproliferative diseases such as Waldenstrom’s macroglobulinemia, non-Hodgkin’s lymphoma, and chronic lymphocytic leukemia^5^	Infections (HCV and hepatitis B) and autoimmune disorders (SLE, Sjogren’s syndrome)^5^^,^^6^
Prevalence	Less common	More common (93% of CG)^8^

*CG*, Cryoglobulinemia; *HCV*, hepatitis C virus; *IgG*, immunoglobulin G; *IgM*, immunoglobulin M; *MC*, mixed CG; *SLE*, systemic lupus erythematosus.

In this case, our patient presented with lower extremity ulcers and purpura that progressed over the course of 4 weeks to necrosis of the bilateral fingers and toes. Necrosis was initially concerning for infection and arteriopathy, which were ruled out with initial negative blood cultures and lack of notable occlusion on Doppler ultrasound or ankle-brachial index, respectively. Work up was continued with additional laboratory work and skin biopsy, which led to a final diagnosis of CG. The patient’s subtype of CG was unable to be determined because of her rapid clinical deterioration and ultimate expiration. Her laboratory work revealed consistencies with each of the types. She had Hepatitis C antibody reactivity, which is typically associated with MC; however, she also had elevated IgG and gamma gap, which are suggestive of a lymphoproliferative pathology, making her condition resemble T1. Additionally, T1 is more associated with ischemia and necrosis rather than the classic triad of MC. On the contrary, she had a benign bone marrow biopsy with only slightly hypercellular aspirate. Regardless, all types of CG may present with skin manifestations, so it is vital that the diagnosis be considered by dermatologists when investigating digital necrosis.

## Conflicts of interest

None disclosed.
